# The effect of dopant and optical micro-cavity on the photoluminescence of Mn-doped ZnSe nanobelts

**DOI:** 10.1186/1556-276X-8-314

**Published:** 2013-07-05

**Authors:** Weichang Zhou, Ruibin Liu, Dongsheng Tang, Bingsuo Zou

**Affiliations:** 1 Key Laboratory of Low-Dimensional Quantum Structures and Quantum Control of Ministry of Education, College of Physics and Information Science, Hunan Normal University, Changsha 410081, China; 2 Beijing Key Laboratory of Nanophotonics and Ultrafine Optoelectronic Systems, School of Materials Science and Engineering, Beijing Institute of Technology, Beijing 100081, China

**Keywords:** Mn dopant, ZnSe nanobelts, Optical micro-cavity, Photoluminescence

## Abstract

Pure and Mn-doped ZnSe nanobelts were synthesized by a convenient thermal evaporation method. Scanning electron microscopy, X-ray powder diffraction, energy dispersive X-ray spectroscopy and corresponding element mapping, and transmission electron microscope were used to examine the morphology, phase structure, crystallinity, composition, and growth direction of as-prepared nanobelts. Raman spectra were used to confirm the effective doping of Mn^2+^ into ZnSe nanobelts. Micro-photoluminescence (PL) spectra were used to investigate the emission property of as-prepared samples. A dominant trapped-state emission band is observed in single *ZnSe*_*Mn*_ nanobelt. However, we cannot observe the transition emission of Mn ion in this ZnSe_Mn_ nanobelt, which confirm that Mn powder act as poor dopant. There are weak near-bandgap emission and strong ^4^*T*_1_ → ^6^*A*_1_ transition emission of Mn^2+^ in single ${\text{ZnSe}}_{{\text{MnCl}}_{2}}$ and ${\text{ZnSe}}_{{\mathrm{Mn}(\mathrm{CH}}_{3}\text{COO})}{}_{{}_{2}}$ nanobelt. More interesting, the ^4^*T*_1_ → ^6^*A*_1_ transition emission in ${\text{ZnSe}}_{{\mathrm{Mn}(\mathrm{CH}}_{3}\text{COO})}{}_{{}_{2}}$ nanobelt split into multi-bands. PL mapping of individual splitted sub-bands were carried out to explore the origin of multi-bands. These doped nanobelts with novel multi-bands emission can find application in frequency convertor and wavelength-tunable light emission devices.

## Background

Recently, doped one-dimension (1D) semiconductor nanostructures are especially attractive for their excellent and unique optical and optoelectronic properties [1,2], which were affected greatly by optical micro-cavity and dopant. 1D nanostructures doped with transition metal (such as Cr, Mn, Fe, Co, and Ni), which can find extensive application in spintronics and nanophotonics [3-5], show novel emission and interesting magnetic transport properties. For example, single crystalline Ga_0.95_Mn_0.05_As nanowires show temperature-dependent hopping conduction [6]. Cu-doped Cd_0.84_Zn_0.16_S nanoribbons show four orders of magnitude larger photocurrent than the undoped ones, demonstrating potential application in photoconductors and chemical sensors [7]. The emission of transition metal ion has specific wavelength, such as the emission of manganese (Mn) ion which is located generally at 585 nm. Moreover, 1D nanostructures can confine the coherent transport or transmission of photon to the definite direction, that is, 1D nanostructures can form optical micro-cavity easily and work as effective optical waveguide within a nanometer scale [8]. Recently, there is an increasing research interest on the optical micro-cavity and corresponding multi-mode emission spectra in doped 1D nanostructures [9]. Zou et al. observed multi-mode emission from doped ZnO nanowires due to F-P cavity effect [10]. Multi-mode emission was also observed in In_*x*_Ga_1 - *x*_N superlattice [11]. Except for the inorganic semiconductor nanostructures, organic nanofibers can also act as coherent random laser with multi-mode emission [12]. Recent research shows that the formation of multi-intracavities plays an important role in the multi-mode emission [13]. These multi-intracavities can couple to produce coherent emission. These confined cavities and multi-band emission of 1D nanostructures are affected strongly by synthesis parameter and deliberate doping. The optical properties of 1D nanostructures are sensitive to minute change of crystal quality, crystal defect, and dopant. The latter can introduce defect state and is therefore very important. So, it is necessary to investigate the direct correlation between dopant and optical properties within the nanometer scale.

ZnSe, a direct semiconductor with a bandgap of 2.63 eV at room temperature, shows excellent optical properties and potential application in light emitting diode and laser diode. 1D ZnSe nanostructures possess novel light emission property [14]. Recently, Vugt et al. observed the novel light-matter interaction in ZnSe nanowires, which can be used to tailor waveguide dispersion and speed of propagating light [15]. In this paper, we synthesize three Mn-ZnSe nanobelts using different dopant compounds. Transmission electron microscopy (TEM) and scanning near-field optical microscopy (SNOM) techniques were used to provide simultaneous investigation on the micro-structure and crystallinity, micro-PL spectrum, and mode-selected mapping image. Both near-bandgap emission and trapped-state emission of ZnSe are observed in Mn-ZnSe nanobelts obtained using Mn powder as dopant. However, the Mn ion transition emission cannot be observed in this ZnSe_Mn_ nanobelt. Using manganese chloride (MnCl_2_) as dopant, strong Mn ion transition emission and weak near-bandgap emission are observed. We can also observe the strong Mn ion transition emission and weak near-bandgap emission in the Mn-ZnSe nanobelts obtained using manganese acetate as dopant. More interestingly, the Mn ion transition emission can split into multi-mode emission due to multi-Fabry-Pérot cavity effect in the ${\text{ZnSe}}_{{\mathrm{Mn}(\mathrm{CH}}_{3}\text{COO})}{}_{{}_{2}}$ nanobelt. Raman spectrum was used to confirm the effective doping. These results are helpful in understanding the effect of dopant on the optical micro-cavities and multi-mode emission. These Mn-ZnSe nanostructures can find promising applications in multicolor emitter or wavelength selective photodetector.

## Methods

The 1D Mn-ZnSe nanobelts were synthesized by a simple thermal evaporation method. Commercial grade mixed powder of ZnSe and Mn or MnCl_2_ or manganese acetate (Mn(CH_3_COO)_2_) with a weight ratio of 5:1 was used as source material. The obtained samples were labeled as ZnSe_Mn_, ${\text{ZnSe}}_{{\text{MnCl}}_{2}}$, ${\text{ZnSe}}_{{\mathrm{Mn}(\mathrm{CH}}_{3}\text{COO})}{}_{{}_{2}}$, respectively. The other synthesis processes are similar with our previous report [16]. The evaporation temperature, growth temperature, and growth time are set to 900°C, 600°C, and 45 min, respectively. A yellow product deposited on the silicon wafer after the furnace cools down to room temperature. For comparison, the pure ZnSe nanobelts were also synthesized using ZnSe powder as source material.

XRD (D/max-5000, Rigaku Corporation, Tokyo, Japan), E-SEM (QUANTA 200, FEI, Hillsboro, OR, USA), energy dispersive X-ray spectroscopy (EDS; attached to SEM), and TEM (JEM-3010, JEOL Ltd., Tokyo, Japan) were used to examine the phase structure, crystallinity, and composition of the as-prepared nanobelts. Raman spectroscopy was performed in a confocal microscope (LABRAM-010, HORIBA Ltd., Kyoto, Japan) using He-Ne laser (632.8 nm) as excitation light source. The PL and corresponding mapping were obtained by SNOM (alpha 300 series, WITec GmbH, Ulm, Germany) with He-Cd laser (325 nm) as excitation source at room temperature. In all optical experiments, the excitation signal illuminated perpendicularly onto the sample surface.

## Results and discussion

**Figure 1:**
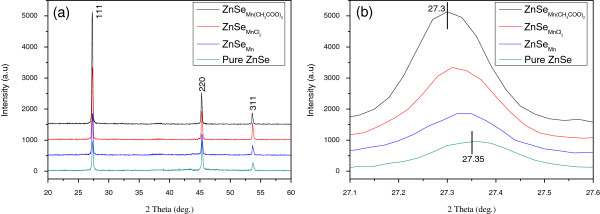
**XRD patterns. (a)** Pure ZnSe, ZnSe_Mn_, ${\text{ZnSe}}_{{\text{MnCl}}_{2}}$, and ${\text{ZnSe}}_{{\mathrm{Mn}(\mathrm{CH}}_{3}\text{COO})}{}_{{}_{2}}$ nanobelts. **(b)** Enlarged (111) diffraction peak of the four samples.

The XRD patterns of pure and doped ZnSe nanobelts are shown in Figure 1. All of the XRD pattern peaks of pure and doped ZnSe nanobelts are in agreement with the standard values (JCPDS card no. 37–1463), see Figure 1a. There are no diffraction peaks of Mn or MnSe in the doped samples. However, the diffraction peaks of doped ZnSe nanobelts shift to lower angle direction compared with pure sample (Figure 1b). Doping can cause a little change to lattice constant. Therefore, the present measurable shift of diffraction peak (about 0.05°) come from doped Mn because of the larger ionic radius of Mn^2+^ (0.80 Å) than that of Zn^2+^ (0.74 Å). Such shift of diffraction peak can also be observed in other doped nanostructures [17-19]. Therefore, manganese can diffuse and dope into ZnSe nanobelts efficiently when MnCl_2_ or Mn(CH_3_COO)_2_ were used as dopants.

**Figure 2:**
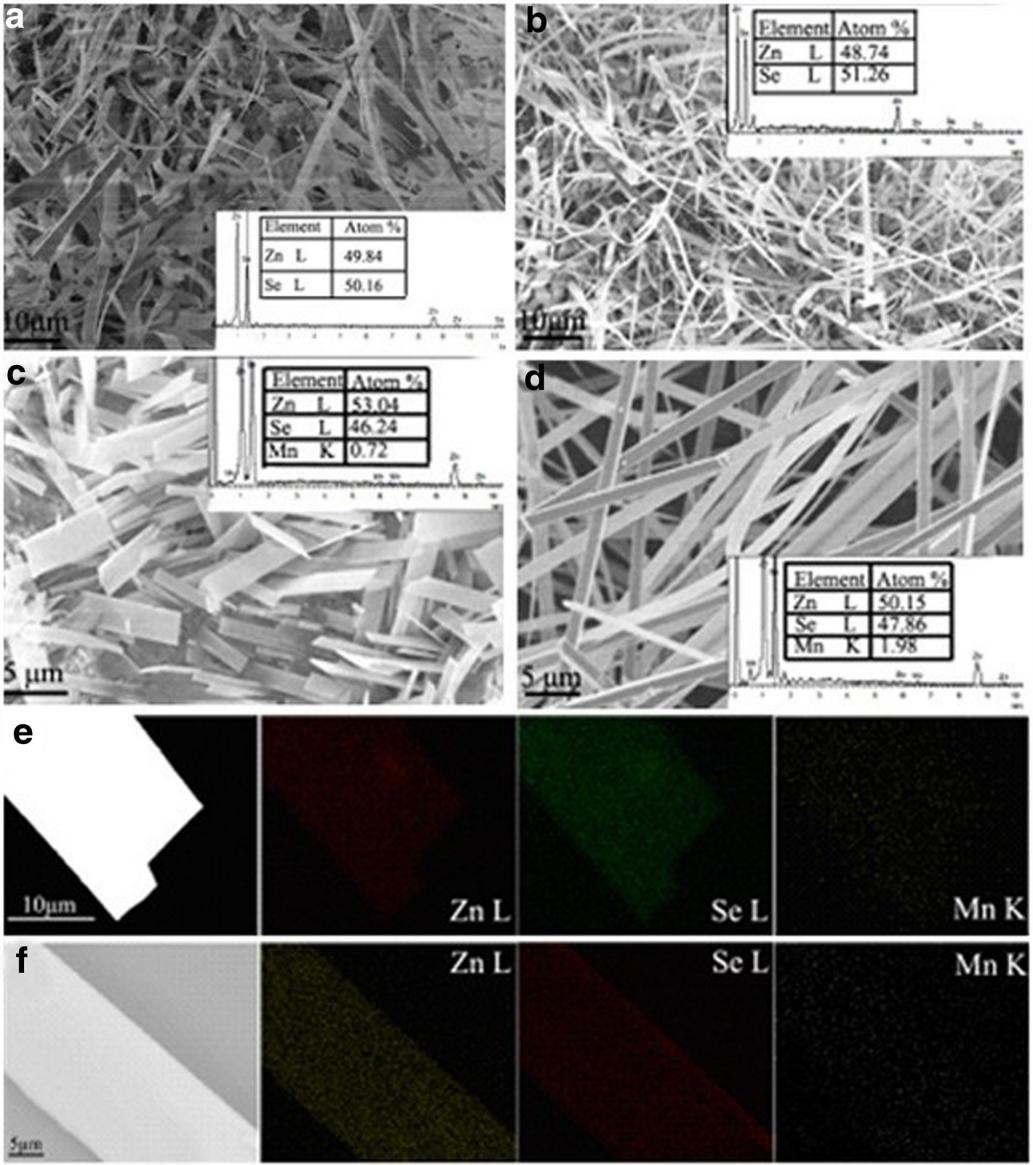
**SEM images and corresponding EDS and element mapping. (a)** to **(d)** Pure ZnSe, ZnSe_Mn_, ${\text{ZnSe}}_{{\text{MnCl}}_{2}}$, and ${\text{ZnSe}}_{{\mathrm{Mn}(\mathrm{CH}}_{3}\text{COO})}{}_{{}_{2}}$ nanobelts, respectively. The insets are the corresponding EDS images. **(e)** to **(f)** Element mapping of single ${\text{ZnSe}}_{{\text{MnCl}}_{2}}$ cand ${\text{ZnSe}}_{{\mathrm{Mn}(\mathrm{CH}}_{3}\text{COO})}{}_{{}_{2}}$ nanobelts, respectively.

Figure 2a is a SEM image of pure ZnSe nanobelts, which deposited on the Si substrate randomly. The nanobelts have a length of hundreds of micro-meter, width of several micro-meter, and thickness of tens of nanometer. EDS (inset of Figure 2a) shows only Zn and Se elements (Si comes from the substrate). The atomic ratio of Zn to Se approaches to 1, demonstrating that pure ZnSe is stoichiometric. Figure 2b,c,d shows the SEM images of doped ZnSe nanobelts obtained using Mn, MnCl_2_, Mn(CH_3_COO)_2_ as dopants. The belt-like morphology of ZnSe_Mn_ is similar with that of pure ZnSe but shows a little difference from those of ${\text{ZnSe}}_{{\text{MnCl}}_{2}}$ and ${\text{ZnSe}}_{{\mathrm{Mn}(\mathrm{CH}}_{3}\text{COO})}{}_{{}_{2}}$. The insets of Figure 2b,c,d are the corresponding EDS images. We cannot detect the Mn element, and the ratio between Zn and Se deviates a little from 1 in ZnSe_Mn_ nanobelts. The dopant concentrations are 0.72% and 1.98% in ${\text{ZnSe}}_{{\text{MnCl}}_{2}}$${\text{ZnSe}}_{{\text{MnCl}}_{2}}$ and ${\text{ZnSe}}_{{\mathrm{Mn}(\mathrm{CH}}_{3}\text{COO})}{}_{{}_{2}}$ nanobelts, respectively. Mn powder is hard to be evaporated due to its high melting point. Therefore, little manganese can dope into the ZnSe nanobelts under the present evaporation temperature when Mn powder was used as the dopant. MnCl_2_ and Mn(CH_3_COO)_2_ have low melting points and are easy to be evaporated. So, manganese can dope into the ZnSe nanobelts effectively when MnCl_2_ or Mn(CH_3_COO)_2_ were used as dopants. The MnCl_2_ and Mn(CH_3_COO)_2_ were usually used as dopants in other semiconductor nanostructures [16,17]. We mapped the elements to detect the distribution of Mn dopant in the nanobelt. Figure 2e shows the EDS mapping of ${\text{ZnSe}}_{{\text{MnCl}}_{2}}$ nanobelt. The mapping profiles show that Mn, Zn, and Se elements distributed homogeneously within the nanobelt. Figure 2f is the EDS mapping of ${\text{ZnSe}}_{{\mathrm{Mn}(\mathrm{CH}}_{3}\text{COO})}{}_{{}_{2}}$ nanobelt, which shows that the distribution of Mn element is inhomogeneous. The minute inhomogeneous distribution of Mn can affect the optical property of the ${\text{ZnSe}}_{{\mathrm{Mn}(\mathrm{CH}}_{3}\text{COO})}{}_{{}_{2}}$ nanobelt greatly.

**Figure 3:**
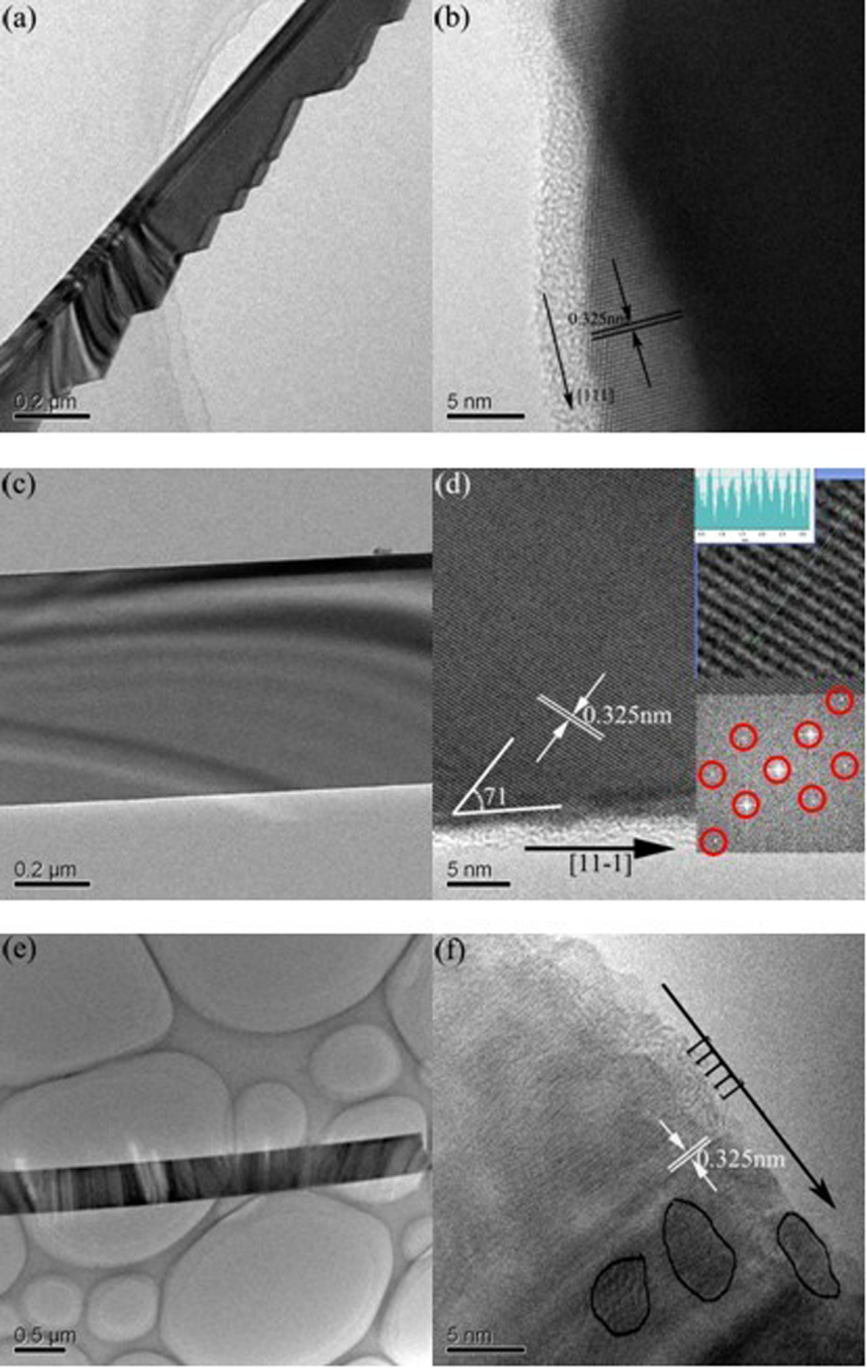
**TEM and HRTEM images. (a)** and **(b)** Single ZnSe_Mn_ nanobelt. **(c)** and **(d)** Single ${\text{ZnSe}}_{{\text{MnCl}}_{2}}$ nanobelt. Insets in **(d)** are the calculated lattice fringe image and FFT. **(e)** and **(f)** Single ${\text{ZnSe}}_{{\mathrm{Mn}(\mathrm{CH}}_{3}\text{COO})}{}_{{}_{2}}$ nanobelt.

Further characterization of these doped ZnSe nanobelt is performed by means of TEM operating at 300 kV. High-resolution TEM (HRTEM) can be used to describe the crystal quality and growth direction. Figure 3a is a TEM image of a ZnSe_Mn_ nanobelt. The morphology and size were consistent with those observed using SEM. Figure 3b is the corresponding HRTEM image. The well-resolved lattice fringes confirmed the single crystalline structure. The measured lattice fringe of 0.325 nm corresponds to the inter-planar distance of (111) plane as known from the bulk ZnSe crystal. Therefore, the growth direction of ZnSe_Mn_ nanobelt is designated to be [111]. The result also confirmed the fact that (111) is the most densely packed facet for fcc structure and is thus the most favorable facet for growth. Figure 3c is a TEM image of ${\text{ZnSe}}_{{\text{MnCl}}_{2}}$ nanobelt. Figure 3d is the corresponding HRTEM image. The ${\text{ZnSe}}_{{\text{MnCl}}_{2}}$ nanobelt shows a single crystalline structure (see the fast Fourier transform (FFT) image in the inset of Figure 3d). The measured lattice fringe is 0.325 nm. The angle between the lattice plane and the axis direction of the nanobelt is 71° (see in Figure 3d). Therefore, the growth direction of the nanobelt can also be designate to be part of the <111> family directions. Figure 3e is a TEM image of the ${\text{ZnSe}}_{{\mathrm{Mn}(\mathrm{CH}}_{3}\text{COO})}{}_{{}_{2}}$ nanobelt. Figure 3f is the corresponding HRTEM image. Similar with ${\text{ZnSe}}_{{\text{MnCl}}_{2}}$ nanobelt, the ${\text{ZnSe}}_{{\mathrm{Mn}(\mathrm{CH}}_{3}\text{COO})}{}_{{}_{2}}$ nanobelt also shows a single crystalline nature and [111] growth direction. The HRTEM also indicates that there are a lot of defect states and impurities in the ${\text{ZnSe}}_{{\mathrm{Mn}(\mathrm{CH}}_{3}\text{COO})}{}_{{}_{2}}$ nanobelt (see the labeled cycle zone in Figure 3f).

**Figure 4:**
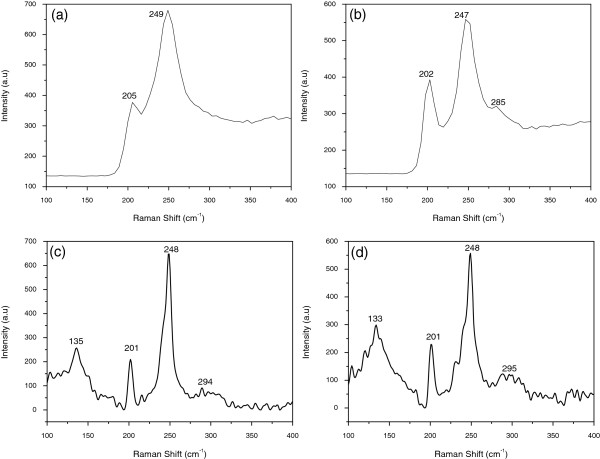
**Raman spectra. (a)** Pure ZnSe, **(b)** ZnSe_Mn_, **(c)**${\text{ZnSe}}_{{\text{MnCl}}_{2}}$, and **(d)**${\text{ZnSe}}_{{\mathrm{Mn}(\mathrm{CH}}_{3}\text{COO})}{}_{{}_{2}}$ nanobelt, respectively.

Raman spectroscopy can provide abundant structure information and is powerful for fast and non-destructive detection of dopant. Figure 4 shows the micro-Raman spectra of single pure and doped ZnSe nanobelt at room temperature. In the Raman spectrum of the pure ZnSe nanobelt (Figure 4a), the peaks at 205 and 249 cm^-1^ can be assigned to TO and LO modes of zinc blende ZnSe crystal, respectively [16]. Figure 4b is the Raman spectrum of the ZnSe_Mn_ nanobelt. Besides the LO and TO vibration modes of ZnSe, there is another mode at 285 cm^-1^ with weak intensity, which related to the defect state (stacking fault) in the doped ZnSe [20]. Figure 4c is the Raman spectrum of ${\text{ZnSe}}_{{\text{MnCl}}_{2}}$ nanobelt. Besides the 201, 248, and 294 cm^-1^ vibration modes, there is another mode at 135 cm^-1^ which is not the intrinsic mode of ZnSe. The 135 cm^-1^ mode can be assigned to the TO impurity vibration modes of MnSe [21]. The presence of impurity vibration modes of MnSe confirms that Mn can dope into ZnSe nanobelts effectively with MnCl_2_ as dopant in the present synthesis parameters. However, the absence of impurity vibration modes of MnSe in ZnSe_Mn_ nanobelt demonstrates that the concentration of Mn^2+^ is too low, and the Mn powder is not the appropriate dopant. The vibration modes of the ${\text{ZnSe}}_{{\mathrm{Mn}(\mathrm{CH}}_{3}\text{COO})}{}_{{}_{2}}$ nanobelt are almost the same with those of the ${\text{ZnSe}}_{{\text{MnCl}}_{2}}$ nanobelt (Figure 4d). The difference of these two Raman spectra is that the intensity ratio of ZnSe to MnSe mode is larger in the ${\text{ZnSe}}_{{\mathrm{Mn}(\mathrm{CH}}_{3}\text{COO})}{}_{{}_{2}}$ nanobelt. The four Raman spectra indicate varied vibration modes, which were affected by the dopant in the Mn-ZnSe nanobelt.

**Figure 5:**
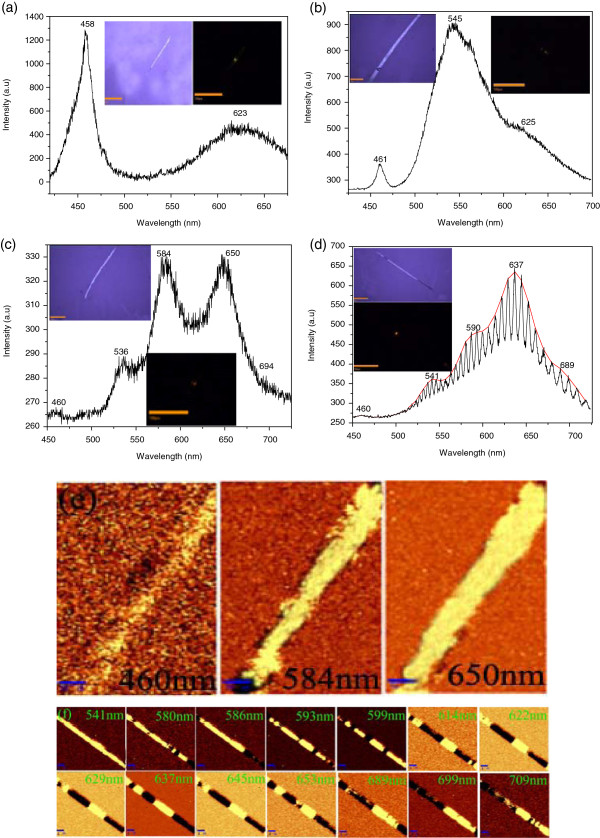
**PL spectra and corresponding emission mapping images. (a)** Pure ZnSe, **(b)** ZnSe_Mn_, **(c)**, ${\text{ZnSe}}_{{\text{MnCl}}_{2}}$, and **(d)**${\text{ZnSe}}_{{\mathrm{Mn}(\mathrm{CH}}_{3}\text{COO})}{}_{{}_{2}}$ nanobelt, respectively. The insets are the corresponding bright-field optical and dark-field emission images. The red curve in **(d)** is the fitted PL spectrum. **(e)** The PL of each individual emission band in **(c)**. **(f)** PL mapping images of individual emission sub-band in **(d)**. The scale is 4 μm.

We studied further the luminescence properties of the as-synthesized Mn-ZnSe nanobelts by commercial SNOM. The insets of Figure 5a are bright-field optical and dark-field emission images of a single representative pure ZnSe nanobelt under the excitation of He-Cd laser (325 nm). The emission is strong at the excitation spot. Figure 5a is the corresponding far-field PL spectrum. The band at 458 nm comes from the near-band edge emission of ZnSe, while the broad emission band at lower energy between 575 and 675 nm is attributed to the trapped-state emission [16]. Trapped-state and dangling bond, such as Zn vacancy and interstitial state, are easy to form in nanostructures due to the reducing size. Therefore, the trapped-state emission is usually observed even in pure nanostructures [22]. The insets of Figure 5b are the bright-field optical and dark-field emission images of a single ZnSe_Mn_ nanobelt. Figure 5b is a corresponding far-field PL spectrum. We can observe a near-band edge emission of ZnSe with low intensity located at 461 nm and the trapped-state emission at 625 nm. There is another strong emission band at 545 nm, which can be explained by the dislocation, stacking faults, and nonstoichiometric defects, as reported in reference [23-25]. We cannot observe the Mn ion emission (such as ^4^*T*_1_ → ^6^*A*_1_ transition emission at 585 nm) which demonstrates that the Mn concentration is too low or there is no Mn doping into the ZnSe_Mn_ nanobelt. The insets of Figure 5c are the bright-field optical and dark-field emission images of ${\text{ZnSe}}_{{\text{MnCl}}_{2}}$ nanobelt. Figure 5c is the corresponding far-field PL spectrum. Except for the weak near-bandgap emission and defect state emissions at 460 and 536 nm, there are two strong emission bands at 584 and 650 nm. The 584-nm band corresponds to d-d (^4^*T*_1_ → ^6^*A*_1_) transition emission of tetrahedral coordinated Mn^2+^ states [26]. The 650-nm band is from the Mn-Mn emission centers, which is similar with the phenomenon of the Mn dimers [27,28]. The Mn-Mn emission only occurs when the Mn dopant concentration is high enough [29]. There is another weak emission band at 694 nm, which is believed to originate from the Mn^2+^ ions at the distorted tetrahedral sites or the octahedral sites, due to the high Mn content [30,31]. Manganese ions on such lattice sites show a different crystal-field splitting between the states of 3*d* orbitals, and then a red-shifted emission band is observed [32]. The appearance of the Mn^2+^ emission demonstrates the efficient doping of Mn^2+^ ion into the ZnSe crystal. We further carried out PL mapping of each individual emission band to explore the distribution of Mn^2+^ ions (Figure 5e). We can see that the distribution of near-band edge emission and Mn^2+^ ion emission is homogeneous in the whole nanobelt (see in Figure 5c). Therefore, the Mn^2+^ ions distribute homogeneously in the nanobelt, consistent with the EDS mapping result. The insets of Figure 5d are the bright-field optical and dark-field emission images of the ${\text{ZnSe}}_{{\mathrm{Mn}(\mathrm{CH}}_{3}\text{COO})}{}_{{}_{2}}$ nanobelt. A portion of the *in situ* emission propagated through the nanobelt and emitted at the opposite end, indicating that the nanobelt can act as an effective optical waveguide. Figure 5d is the corresponding far-field PL spectrum, which contains a near-band edge emission and a broad emission band between 525 and 725 nm. Similar to the PL spectrum of ${\text{ZnSe}}_{{\text{MnCl}}_{2}}$ nanobelt, the broad emission contains four bands: 541, 590, 637, and 689 nm (see the fitted red curve in Figure 5d). Therefore, the Mn^2+^ ion efficiently doped into the ZnSe matrix crystal with ${\text{ZnSe}}_{{\mathrm{Mn}(\mathrm{CH}}_{3}\text{COO})}{}_{{}_{2}}$ as dopant. Moreover, in contrast to the reported Mn^2+^ transition emission (see the PL of the ${\text{ZnSe}}_{{\text{MnCl}}_{2}}$ nanobelt), the current Mn^2+^ emission band splits into many narrow sub-bands, that is, multi-mode emission. The PL mapping is carried out for individual sub-bands to explore the origin of the multi-mode emission and photon propagation process in the nanobelt (Figure 5f). We can see that the near-band edge emission distributes in the whole nanobelt. In contrast, the mapping images of the Mn^2+^ ion emission sub-bands show irregular light intensity distribution along the nanobelt (the bright and dark regions represent the maximum and minimum intensities of emission, respectively). Moreover, there is slight modification between these Mn^2+^ ion emission mappings, such as it is a bright region at the end of 599 nm band, while it is dark for 637-nm band at the same position. This is due to the cavity mode selection within the belt. The mapping images indicate that there are several optical micro-cavities within the single nanobelt. Usually, the two end facets act as reflecting mirrors to form one Fabry-Pérot cavity in 1D nanostructures. However, multi-cavities can emerge in single doped 1D nanostructure when a dopant with varied refractive indexes is incorporated into the matrix [13,16]. In the HRTEM image (Figure 3f), we can clearly see some impurity and defect sites possibly related to the Mn dopant in the ${\text{ZnSe}}_{{\mathrm{Mn}(\mathrm{CH}}_{3}\text{COO})}{}_{{}_{2}}$ nanobelt. When the nanobelt was excited, a large number of photons propagate along the axis, in which some were absorbed, some were reflected or scattered by high refractive index domain, and some others passed through the segment boundary. These reflected photons propagate to another boundary and resonate at the boundary zones. So, different emission lines were selected to be observed in a single nanobelt. Combining the mapping images and multi-modes spectra, we can calculate the sub-cavity length *L* using the formula: Δ, where *n* is the refractive index (*n* = 2.67 for ZnSe), *λ*_1_ and *λ*_2_ are the resonant wavelengths, and *Δλ* is the mode spacing [16]. The calculated cavity lengths of the adjacent bands are 9 to 11 μm, which are much shorter than the actual length of the nanobelt, but very close to the lengths of bright region in the mapping images. Therefore, the photon oscillation within a single nanobelt does not always happen between the two ends, but rather in the smaller inner segments and then result in multi-cavity and multi-mode emissions. Such *in situ* PL spectrum and mapping indicate strong localization and oscillation of photon propagation along the longitudinal axis. This behavior is a typical coupled optical multi-cavity.

**Figure 6:**
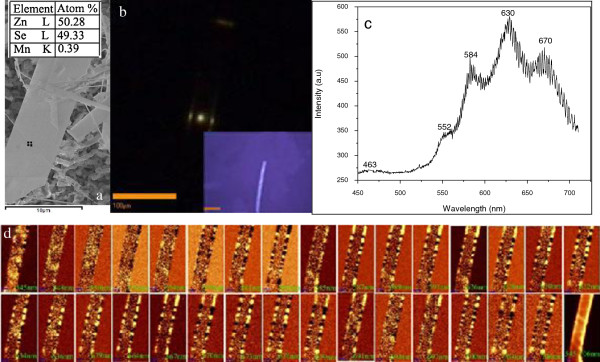
**Characterization of another**${\mathrm{ZnSe}}_{{\mathrm{Mn}(\mathrm{CH}}_{3}\mathrm{COO})}{}_{{}_{2}}$**nanobelt with low Mn**^**2+ **^**concentration (0.39%). (a)** SEM image and EDS. **(b)** Dark-field emission image. The inset is the corresponding bright-field optical image. **(c)** The corresponding PL spectrum. **(d)** The corresponding PL mapping images of individual emission sub-bands. The scale is 10 μm.

The growth conditions can be adjusted to obtain another ${\text{ZnSe}}_{{\mathrm{Mn}(\mathrm{CH}}_{3}\text{COO})}{}_{{}_{2}}$ nanobelt. Figure 6a is the SEM image and EDS of the ${\text{ZnSe}}_{{\mathrm{Mn}(\mathrm{CH}}_{3}\text{COO})}{}_{{}_{2}}$ nanobelt with lower Mn concentration (0.39%). Figure 6b is the dark-field emission image of single ${\text{ZnSe}}_{{\mathrm{Mn}(\mathrm{CH}}_{3}\text{COO})}{}_{{}_{2}}$ nanobelt with 0.39% Mn content, which also shows the optical waveguide characteristic. The inset is the corresponding bright-field optical image. Figure 6c is the corresponding far-field PL spectrum. The PL spectrum contains near-band edge emission of ZnSe with weak intensity and transition emission of Mn^2+^ with strong intensity. Compared with Figure 5d, the split of Mn^2+^ emission in Figure 6c is not evident. We can distinguish ambiguously that the Mn^2+^ emission split into many narrow sub-bands with a smaller periodic span (about 2 nm). The PL mapping is carried out for individual sub-bands to see if there are integrated multi-cavities in the nanobelt (Figure 6d). We can see that the band of 552 nm distributes homogeneously in the whole nanobelt. The sub-bands of 584, 630 and 670 nm distribute almost at two sides of the nanobelt. The excited photon emits at the side and end of the nanobelt usually after scattering at the boundary many times [33]. The optical multi-cavity phenomenon is not evident, although it still exists in the nanobelt due to the incontinuous emission intensity distribution at the two sides. The reduced Mn content can reduce the impurity and trapped state in the nanobelt and then affect the cavity quality greatly. Therefore, both dopant and micro-cavity play an important role in the multi-modes emission.

## Conclusions

We synthesized pure and Mn-doped ZnSe nanobelts successfully using thermal evaporation method. Mn can dope effectively into ZnSe crystal when MnCl_2_ or Mn(CH_3_COO)_2_ were used as dopants in the source material. EDS mapping indicates that the distribution of Mn is inhomogeneous in the ${\text{ZnSe}}_{{\mathrm{Mn}(\mathrm{CH}}_{3}\text{COO})}{}_{{}_{2}}$ nanobelt. All of these doped nanobelts grew along the <111> direction. HRTEM demonstrates that there are a lot of defect states in the ${\text{ZnSe}}_{{\mathrm{Mn}(\mathrm{CH}}_{3}\text{COO})}{}_{{}_{2}}$ nanobelt. Raman spectra confirm that Mn^2+^ was doped into ${\text{ZnSe}}_{{\text{MnCl}}_{2}}$ and ${\text{ZnSe}}_{{\mathrm{Mn}(\mathrm{CH}}_{3}\text{COO})}{}_{{}_{2}}$ nanobelts successfully. The optical properties are affected strongly by the concentration and spatial distribution of the dopant. Optical micro-cavity also plays an important role to the emission property. Nanobelt shows strong ^4^*T*_1_ → ^6^*A*_1_ transition emission of Mn^2+^. However, the ^4^*T*_1_ → ^6^*A*_1_ transition emission of Mn^2+^ in ${\text{ZnSe}}_{{\mathrm{Mn}(\mathrm{CH}}_{3}\text{COO})}{}_{{}_{2}}$ nanobelt splits into many narrow sub-bands due to the formation of integrated multi-Fabry-Pérot cavities, which can couple to produce coherent emission with selected wavelength and cavity mode. PL mapping confirms that there are several micro-cavities in the single ${\text{ZnSe}}_{{\mathrm{Mn}(\mathrm{CH}}_{3}\text{COO})}{}_{{}_{2}}$ nanobelt. Such doped nanobelts with integrated multi-micro-cavities and modulated emission wavelength can be optimized to fabricate nanophotonic devices and quantum coherent modulators.

## Competing interests

The authors declare that they have no competing interests.

## Authors' contributions

WZ prepared the manuscript and carried out the experiment. RL helped in the technical support for the PL measurements. DT and BZ helped in the discussion and analysis of the experimental results. All authors read and approved the final manuscript.

## Authors' information

WZ got his PhD degree in 2010. He is an assistant professor now. RL is an associate professor. DT and BZ are professors.

## References

[B1] Liu C Sun JW Tang JY Yang PD Zn-doped p-type gallium phosphide nanowire photocathodes from a surfactant-free solution synthesis Nano Lett 2012 8 5407 5411 10.1021/nl3028729 23025657

[B2] Nie B Luo LB Chen JJ Hu JG Wu CY Wang L Yu YQ Zhu ZF Jie JS Fabrication of p-type ZnSe:Sb nanowires for high-performance ultraviolet light photodetector application Nanotechnology 2013 8 095603 10.1088/0957-4484/24/9/095603 23403941

[B3] Zeng YJ Pereira LMC Menghini M Temst K Vantomme A Locquet JP Haesendonck CV Tuning quantum corrections and magnetoresistance in ZnO nanowires by ion implantation Nano Lett 2012 8 666 672 10.1021/nl2034656 22214218

[B4] Feng GY Yang C Zhou SH Nanocrystalline Cr^2+^-doped ZnSe nanowires laser Nano Lett 2013 8 272 275 10.1021/nl304066h 23256521

[B5] López I Nogales E Méndez B Javier P Influence of Sn and Cr doping on morphology and luminescence of thermally grown Ga_2_O_3_ nanowires J Phys Chem C 2013 8 3036 3045 10.1021/jp3093989

[B6] Paschoal W Jr Kumar S Borschel C Wu P Canali CM Ronning C Samuelson L Pettersson H Hopping conduction in Mn ion-implanted GaAs nanowires Nano Lett 2012 8 4838 4842 10.1021/nl302318f 22889471

[B7] Lui TY Zapien JA Tang H Ma DDD Liu YK Lee CS Lee ST Shi SL Xu SJ Photoluminescence and photoconductivity properties of copper-doped Cd_1-*x*_Zn_*x*_S nanoribbons Nanotechnology 2006 8 5935 10.1088/0957-4484/17/24/006

[B8] Huang MH Mao S Feick H Yan HQ Wu YY Kind H Weber E Russo R Yang PD Room-temperature ultraviolet nanowire nanolasers Science 2001 8 1897 1899 10.1126/science.1060367 11397941

[B9] Pauzauskie PJ Yang PD Nanowire photonics Mater Today 2006 8 36 45

[B10] Zou BS Liu RB Wang FF Pan AL Cao L Wang ZL Lasing mechanism of ZnO nanowires/nanobelts at room temperature J Phys Chem B 2006 8 12865 12873 10.1021/jp061357d 16805584

[B11] Qian F Li Y Gradecak S Park HG Dong Y Ding Y Wang ZL Lieber CM Multi-quantum-well nanowire heterostructures for wavelength-controlled lasers Nature Mater 2008 8 701 706 10.1038/nmat2253 18711385

[B12] Quochi F Random lasers based on organic epitaxial nanofibers J Opt 2010 8 024003 10.1088/2040-8978/12/2/024003

[B13] Li Y Dai GZ Zhou CJ Zhang QL Wan Q Fu LM Zhang JP Liu RB Cao CB Pan AL Zhang YH Zou BS Formation and optical properties of ZnO:ZnFe_2_O_4_ superlattice microwires Nano Res 2010 8 326 338 10.1007/s12274-010-1036-y

[B14] Saxena A Yang SX Philipose U Ruda HE Excitonic and pair-related photoluminescence in ZnSe nanowires J Appl Phys 2008 8 053109 10.1063/1.2885729

[B15] Vugt LK Zhang B Piccione B Spector AA Agarwal R Size-dependent waveguide dispersion in nanowire optical cavities: slowed light and dispersionless guiding Nano Lett 2009 8 1684 1688 10.1021/nl900371r 19265428

[B16] Zhou WC Liu RB Tang DS Wang XX Fan HM Pan AL Zhang QL Wan Q Zou BS Luminescence and local photonic confinement of single ZnSe:Mn nanostructure and the shape dependent lasing behavior Nanotechnology 2013 8 055201 10.1088/0957-4484/24/5/055201 23306604

[B17] Lee JY Kim DS Kang JH Yoon SW Lee H Park J Novel Zn_1-*x*_Mn_*x*_Se (*x*=0.1-0.4) one-dimensional nanostructures: nanowires, zigzagged nanobelts, and toothed nanosaws J Phys Chem B 2006 8 25869 25874 10.1021/jp065749w 17181234

[B18] Kang JW Choi YS Choe M Kim NY Lee T Kim BJ Tu CW Park SJ Electrical and structural properties of antimony-doped p-type ZnO nanorods with self-corrugated surfaces Nanotechnology 2012 8 495712 10.1088/0957-4484/23/49/495712 23154405

[B19] Suh M Meyyappan M Ju S The effect of Ga content on In_2*x*_Ga_2-2*x*_O_3_ nanowire transistor characteristics Nanotechnology 2012 8 305203 10.1088/0957-4484/23/30/305203 22750916

[B20] Wang FF Zhang ZH Liu RB Wang X Zhu X Pan AL Zou BS Structure and stimulated emission of ZnSe nanoribbons grown by thermal evaporation Nanotechnology 2007 8 305705 10.1088/0957-4484/18/30/305705

[B21] Popović ZV Milutinović A Far-infrared reflectivity and Raman scattering study of *α*-MnSe Phys Rev B 2006 8 155203

[B22] Jiang Y Meng XM Yiu WC Liu J Ding JX Lee CS Lee ST Zinc selenide nanoribbons and nanowires J Phys Chem B 2004 8 2784 2787 10.1021/jp035595+

[B23] Leung YP Wallace CHC Markov I Pang GKH Ong HC Yuk TI Synthesis of wurtzite ZnSe nanorings by thermal evaporation Appl Phys Lett 2006 8 183110 10.1063/1.2200155

[B24] Philipose U Xu T Yang S Sun P Ruda HE Wang YQ Kavanagh KL Enhancement of band edge luminescence in ZnSe nanowires J Appl Phys 2006 8 084316 10.1063/1.2362930

[B25] Panda AB Acharya S Efrima S Ultranarrow ZnSe nanorods and nanowires: structure, spectroscopy, and one-dimensional properties Adv Mater 2005 8 2471 2474 10.1002/adma.200500551

[B26] Na CW Han DS Kim DS Kang YJ Lee JY Park J Oh DK Kim KS Kim D Photoluminescence of Cd_1-*x*_Mn_*x*_S (*x*≤0.3) nanowires J Phys Chem B 2006 8 6699 6704 10.1021/jp060224p 16570975

[B27] Chen W Sammynaiken R Huang Y Malm JO Wallenberg R Bovin JO Zwiller V Kotov NA Crystal field, phonon coupling and emission shift of Mn^2+^ in ZnS:Mn nanoparticles J Appl Phys 2001 8 1120 10.1063/1.1332795

[B28] Liu QH Sun ZH Yan WS Zhong WJ Pan ZY Hao LY Wei SQ Anomalous magnetic behavior of Mn-Mn dimers in the dilute magnetic semiconductor (Ga, Mn)N Phys Rev B 2007 8 245210

[B29] Pradhan N Peng XG Efficient and color-tunable Mn-doped ZnSe nanocrystal emitters: control of optical performance via greener synthetic chemistry J Am Chem Soc 2007 8 3339 3347 10.1021/ja068360v 17311383

[B30] Goede O Thong DD Energy transfer processes in (Zn, Mn)S mixed crystals Phys Status Solidi B 1984 8 343 353 10.1002/pssb.2221240137

[B31] Kim DS Cho YJ Park J Yoon J Jo Y Jung MH (Mn, Zn) Co-doped CdS nanowires J Phys Chem C 2007 8 10861 10868

[B32] Barglik-Chory C Remenyi C Dem C Schmitt M Kiefer W Gould C Rüster C Schmidt G Hofmann DM Pfistererd D Müller G Synthesis and characterization of manganese-doped CdS nanoparticles Phys Chem Chem Phys 2003 8 1639 1643 10.1039/b300343d

[B33] Vugt LKV Rühle S Ravindran P Gerritsen HC Kuipers L Vanmaekelbergh D Exciton polaritons confined in a ZnO nanowire cavity Phys Rev Lett 2006 8 147401 17155289 10.1103/PhysRevLett.97.147401

